# Enhancement of Insulin/PI3K/Akt Signaling Pathway and Modulation of Gut Microbiome by Probiotics Fermentation Technology, a Kefir Grain Product, in Sporadic Alzheimer’s Disease Model in Mice

**DOI:** 10.3389/fphar.2021.666502

**Published:** 2021-07-23

**Authors:** Nesrine S. El Sayed, Esraa A. Kandil, Mamdooh H. Ghoneum

**Affiliations:** ^1^ Department of Pharmacology and Toxicology, Cairo University, Cairo, Egypt; ^2^ Department of Surgery, Charles R. Drew University of Medicine and Science, Los Angeles, CA, United States

**Keywords:** metabolic syndrome, insulin signaling, alzheimer's disease, PI3/Akt pathway, PFT

## Abstract

Sporadic Alzheimer’s disease (AD) is the most common neurodegenerative disorder with cognitive dysfunction. Remarkably, alteration in the gut microbiome and resultant insulin resistance has been shown to be connected to metabolic syndrome, the crucial risk factor for AD, and also to be implicated in AD pathogenesis. Thus, this study, we assessed the efficiency of probiotics fermentation technology (PFT), a kefir product, in enhancing insulin signaling via modulation of gut microbiota to halt the development of AD. We also compared its effectiveness to that of pioglitazone, an insulin sensitizer that has been confirmed to substantially treat AD. AD was induced in mice by a single injection of intracerebroventricular streptozotocin (STZ; 3 mg/kg). PFT (100, 200, 400 mg/kg) and pioglitazone (30 mg/kg) were administered orally for 3 weeks. Behavioral tests were conducted to assess cognitive function, and hippocampal levels of acetylcholine (Ach) and β-amyloid (Aβ_1–42_) protein were assessed along with histological examination. Moreover, the expression of the insulin receptor, insulin degrading enzyme (IDE), and the phosphorylated forms of phosphoinositide 3-kinase (PI3K), protein kinase B (Akt), glycogen synthase kinase-3β (GSK-3β), mammalian target of rapamycin (mTOR), and tau were detected. Furthermore, oxidative stress and inflammatory biomarkers were estimated. Treatment with PFT reversed STZ-induced neurodegeneration and cognitive impairment, enhanced hippocampal Ach levels, and reduced Aβ_1–42_ levels after restoration of IDE activity. PFT also improved insulin signaling, as evidenced by upregulation of insulin receptor expression and activation of PI3K/Akt signaling with subsequent suppression of GSK-3β and mTOR signaling, which result in the downregulation of hyperphosphorylated tau. Moreover, PFT significantly diminished oxidative stress and inflammation induced by STZ. These potential effects were parallel to those produced by pioglitazone. Therefore, PFT targets multiple mechanisms incorporated in the pathogenesis of AD and hence might be a beneficial therapy for AD.

## Introduction

Sporadic Alzheimer's disease (AD) is the major form of cognitive impairment and dementia (Chakrabarti et al., 2015; Jagust, 2018). The hallmark of the disease is the aggregation of amyloid plaques, which are mainly formed from β-amyloid (Aβ_1–42_) protein, and the accumulation of neurofibrillary tangles in the neurons, which are primarily composed of phosphorylated tau (p-tau) protein (Xin et al., 2018). Previous studies revealed that insulin resistance, oxidative stress, and inflammation are highly correlated with the pathogenesis of AD (Finder, 2010; Tong et al., 2012). Importantly, metabolic syndrome, which is a combination of obesity, insulin resistance, and hypertriglyceridemia, is greatly implicated in AD pathology (Hishikawa et al., 2016; Tyagi et al., 2020). It is assumed that insulin resistance in metabolic syndrome is the vital cause of this linkage, as impaired insulin signaling contributes to the pathogenesis of AD (Tyagi et al., 2020). Markedly, insulin signaling in the brain has been shown to display a crucial role in learning and memory, besides promoting neuronal development (Butterfield et al., 2014; Gabbouj et al., 2019). However, insulin resistance was shown to be implicated in AD pathogenesis and diminished insulin signaling have been detected in the brains of AD patients (Talbot et al., 2012; Ekblad et al., 2018). In this context, insulin resistance was revealed to promote Aβ secretion and to halt its clearance by decreasing the production of insulin degrading enzyme (IDE), a key enzyme for Aβ degradation, and competing with Aβ for IDE (Zhao et al., 2004; Gabbouj et al., 2019). Moreover, it results in mitochondrial dysfunction with consequent oxidative stress and elevation of advanced glycation end-products (AGE), which are all implicated in AD development (Butterfield et al., 2014). Furthermore, insulin resistance is associated with inflammation, which is a contributing factor in AD neurodegeneration (Fishel et al., 2005). To this end, insulin resistance in metabolic syndrome underlies several pathological processes involved in AD development.

Remarkably, the gut microbiota has been revealed to regulate cognitive function via the microbiota-gut-brain axis. However, its alteration (dysbiosis) has been immensely connected to AD neurodegeneration, since it is implicated in insulin resistance and the generation of inflammatory and oxidative stress states (Zhao et al., 2015; Pistollato et al., 2016; Azm et al., 2018; Angelucci et al., 2019). Importantly, metabolic syndrome has also been demonstrated to be related to dysbiosis (Wang et al., 2020). Thus, the modulation of gut microbiota might be a beneficial strategy for halting the pathophysiological risks implicated in AD development.

Probiotics fermentation technology (PFT) kefir grain product is obtained from kefir, which is a probiotic drink that improves health and is chiefly composed of *Lactobacillus kefiri* (Ghoneum and Gimzewski, 2014). *Lactobacillus kefiri* maintains probiotic bacterial balance and imparts several health benefits together with diminishing insulin resistance, oxidative stress, and inflammation (Carasi et al., 2015; Sun et al., 2020). Several studies revealed safety and tolerability of PFT, in addition to its beneficial effects (Ghoneum and Felo, 2015; Badr El-Din et al., 2020; Ghoneum et al., 2020). Thus, PFT may be effective in treating AD.

Therefore, we conducted this study to discover the efficiency of PFT in attenuating pathological mechanisms involved in AD development. We also aimed to compare the activity of PFT with that of pioglitazone, an insulin sensitizer, which has been confirmed to effectively prevent neurodegeneration and improve cognitive function in an AD animal model (Sato et al., 2011; Mandrekar-Colucci et al., 2012; Searcy et al., 2012).

## Materials and Methods

### Animals

Male albino adult mice (25–30 g) were used in this study. Animals were housed in the animal facility of the Faculty of Pharmacy, Cairo University, Egypt, at a constant temperature (25 ± 2 °C) and humidity level (60 ± 10%) under a 12/12 h light/dark cycle and with free access to food and water. The study was permitted by the Institutional Animal Care and Use Committee of Cairo University (CU-IACUC; permit No. CU-III-F-35-20) and adhered to the Guide for the Care and Use of Laboratory Animals published by the US National Institutes of Health (NIH Publication No. 85-23, revised 2011).

### Chemicals and Drugs

PFT kefir grain product is a combination of an ∼90% heat-killed freeze-dried form of *Lactobacillus kefiri* and ∼2–3% of single bacterial strain (*Lactobacillus kefiri* P-B1) and multiple yeast strains (*Kazachstania turicensis*, *Kazachstania unispora*, and *Kluyveromyces marxianus*) (Ghoneum and Gimzewski, 2014). Positron-emission tomography scans showed that PFT exhibited 99.6% homology with regular kefirs. Huge amounts of yeast strains are found in PFT when it is attained from the Caucasus Mountains, which are removed to obtain optimal kefiri levels. PFT was supplied by Paitos Co. Ltd. (Yokohama, Kanagawa, Japan).

Streptozotocin (STZ) and pioglitazone were purchased from Sigma–Aldrich (St. Louis, MO, United States). Additional chemicals and reagents, if not specified, were also purchased from Sigma–Aldrich Chemical Co.

### Induction of Alzheimer’s Disease

A single STZ injection was administered intracerebroventricularly (ICV) via the freehand method with avoidance of diffusion into the cerebral vein according to previously described procedures (Pelleymounter et al., 2000; Pelleymounter et al., 2002; Warnock, 2007). Briefly, after anesthesia with thiopental (5 mg/kg, intraperitoneally), downward compression was applied above the mouse’s ears to stabilize its head, and the needle was then directly introduced into the lateral ventricle through the skull while observing a symmetrical triangle between the eyes and the skull center to target it. Animals acted normally approximately 1 min after the ICV injection.

### Experimental Design

Mice were randomly divided into six groups consisting of 12 mice each. The study took 21 days. Group I, which served as the sham control group, received a single ICV injection of 0.9% saline, in addition to Tween 80 with 0.9% saline (orally via gavage) daily for 21 consecutive days. Group II, which served as the sporadic AD-model group, received a single ICV injection of STZ (3 mg/kg in 3 µl) dissolved in 0.9% saline (Abdel Rasheed et al., 2018). Groups III to VI received one ICV STZ injection (3 mg/kg), in addition, group III was treated with pioglitazone (30 mg/kg, orally via gavage) suspended in Tween 80 with 0.9% saline 5 h after STZ injection and then daily for a total of 21 doses (Gupta and Gupta, 2012). Groups IV to VI were treated with PFT (100 mg, 200 mg, and 400 mg/kg, orally via gavage, respectively) suspended in Tween 80 with 0.9% saline 5 h after STZ injection and then daily for a total of 21 doses (Maeda et al., 2004). PFT was not administered in normal animals since its safety and tolerability was proved in previous studies (Ghoneum and Felo, 2015; Badr El-Din et al., 2020; Ghoneum et al., 2020) and in order to adhere to the ethical rules for dealing with experimental animals in scientific research in not abusing the animals.

### Behavioral Assessment

We performed behavioral tests 24 h after the administration of the last drug. The less stressful test was performed before the more stressful one, and both tests were carried out during the light cycle to reduce circadian variability.

#### Novel Object Recognition Test

To examine learning and memory, we performed the novel object recognition (NOR) test (Antunes and Biala, 2012). The test is based on the animals’ innate preference for a novel object, in which mice remembering the familiar object will devote more time exploring a novel one. The test was conducted over three successive days. On the first day (habituation), mice were placed into a wooden box (40 cm × 40 cm × 40 cm) individually and permitted to explore the environment (without objects) for 10 min. On the second day (training), mice were placed individually in the middle of the box and given 10 min to visually explore two similar objects placed at the opposite corners of the box. On the third day (test), a novel object was added instead one of the explored objects, and each animal was permitted to explore the objects in the box for 3 min. Exploration was demarcated as directing the animal’s nose to the object at a distance of less than 2 cm, while sitting on the object was not measured as exploration. All objects were cleaned with 70% ethanol after each test to avoid bias due to odors left by previous animals. The test was video recorded, and the following parameters were measured:

Discrimination index: Is the subtraction of the time spent in exploring the novel object and the familiar object divided by the entire time consumed exploring the two objects (this value diverges between −1 and +1, with a negative score in case of spending more time in exploring the familiar object, a zero-score in case of no preference, and a positive score in case of displaying more time discovering the novel object).

Recognition index: Time spent by the animal to explore the novel object as a percentage of the entire time of exploration.

#### Morris Water Maze Test

To inspect spatial memory and learning, we also conducted the Morris water maze (MWM) test (D’Hooge and De Deyn, 2001). A stainless-steel spherical tank (150 cm in diameter and 60 cm in height) was sectioned into four quadrants and loaded with water (25 ± 2 °C) to a depth of 35 cm. A black platform (10 cm in width, 28 cm in height) was situated in the target quadrant, and the tank was loaded with water to 2 cm beneath the platform’s top. The platform was situated in the same place throughout the training and testing trials. During testing, a nontoxic purple dye was added to the water to render it opaque, thus masking the platform. For the first four days, we carried out memory acquisition trials, in which two trials were performed daily (120 s/trial) with 15 min between them. During the trials, each mouse was allowed to find the disguised platform in the target quadrant for 120 s and was thereafter left there for more 20 s, then removed. If the mouse did not find the masked platform in 120 s, it was directed to it and left there for 20 s. The time required by each animal to get to the masked platform was designated as the mean escape latency and used as a learning index. On the fifth day, each mouse was permitted to explore the tank for 60 s after removing the platform. We assessed the time consumed by each animal in the target quadrant, where the disguised platform was formerly situated, and used this time as an indicator of memory.

### Brain Processing

After the behavioral tests, the animals were sacrificed by cervical dislocation under light anesthesia. The brains of the animals of each group were then removed and divided into three sets. Brains from the first set (*n* = 3 per group) were fixed in 10% (v/v) formalin for 24 h to perform histological examination. In the other two sets, the hippocampal tissues were dissected from each brain on an ice-cold glass plate. The hippocampi of the second set (*n* = 6 per group) were homogenized in ice-cold physiological saline (10% w/v), and the homogenate was used to assess the levels of acetylcholine (Ach), Aβ_1−42_, oxidative stress parameters (malondialdehyde [MDA] and glutathione [GSH]), inflammatory markers (phosphorylated nuclear factor kappa beta [p-NF-kB], tumor necrosis factor alpha [TNF-α], interleukin-6 [IL6], and NLRP3), and IDE. In the third set (*n* = 3 per group), hippocampi were used to assess the protein expression of phosphorylated phosphoinositide 3-kinase (p-PI3K), phosphorylated protein kinase B (p-Akt), phosphorylated glycogen synthase kinase 3β (p-GSK-3β), phosphorylated mammalian target of rapamycin (p-mTOR), and p-tau, as well as to determine the gene expression of insulin receptor, receptor for advanced glycation end products (RAGE), and toll-like receptor 4 (TLR4).

### Measured Parameters

#### Determination of Protein Content

The protein content was measured following the method of Lowry et al. (1951).

#### Enzyme-Linked Immunosorbent Assay (ELISA) to Determine the Levels of Ach, Aβ_1–42_, p-NF-κB, TNF-α, IL6, NLRP3, and IDE in the hippocampus

Mouse ELISA kits for Ach (Cat. No. MBS733116), p-NF-κB (Cat. No. MBS2023542), NLRP3 (Cat. No. MBS920134), and IDE (Cat. No. MBS2019335) were bought from Mybiosource (San Diego, CA, United States). Mouse ELISA kits for Aβ_1–42_ (Cat. No. KMB3441), IL6 (Cat. No. KMC0061), and TNF-α (Cat. No. BMS607-3) were purchased from Invitrogen (Carlsbad, CA, United States). We carried out the assessments as described by the manufacturers. The results of Ach and NLRP3 were stated as ng/mg protein, whereas those of Aβ_1–42_, p-NF-κB, TNF-α, IL6, and IDE were stated as pg/mg protein.

#### Colorimetric Determination of hippocampal MDA and GSH

MDA, lipid peroxidation product, and GSH, an antioxidant enzyme, were assessed using commercial colorimetric kits (Biodiagnostics, Cairo, Egypt) in accordance with the manufacturers’ directions.

#### Western Blot Analysis of p-PI3K, p-Akt, p-GSK-3β, p-mTOR, and p-tau Protein Expression in the Hippocampus

Hippocampal tissues were homogenized in ice-cold protein lysis buffer radioimmunoprecipitation assay (RIPA) buffer (9.1 mmol/L dibasic sodium phosphate, 1.7 mmol/L monobasic sodium phosphate, 150 mmol/L sodium chloride, 1% nonidet P-40, 0.5% sodium deoxycholate, 0.1% sodium dodecyl sulfate [pH adjusted to 7.4]). These contained fresh protease and phosphatase inhibitor cocktails, in addition to 1 mmol/L ethylenediaminetetraacetic acid, 1 mmol/L sodium orthovanadate, and 0.2 mmol/L 4-2-aminoethyl benzene sulfonyl fluoride. After determination of protein levels using bicinchoninic acid protein kit (Thermo Fisher Scientific, Waltham, MA, United States), equal amount of protein from each sample (10 µg) was separated by sodium dodecyl sulfate polyacrylamide gel electrophoresis and then moved to a nitrocellulose membrane (Amersham Bioscience, Piscataway, NJ, United States), which was then blocked via immersion in 5% skimmed milk overnight. Subsequently, the membranes were incubated with primary antibodies against p-PI3K (1:500, Cat. No. ab182651), p-Akt (0.005 µg/ml, Cat. No. ab278559), p-GSK3β (1 µg/ml, Cat. No. ab107166), p-mTOR (1:1000, Cat. No. ab109268), and p-tau (1:10,000, Cat. No. ab109390) (Abcam, Cambridge, MA, United States) overnight at 4 °C on a roller shaker. The membranes were then incubated with peroxidase-conjugated secondary antibodies. Blots were envisioned using the enhanced chemiluminescence detection reagents (Amersham Biosciences, Arlington Heights, IL, United States) and quantified relative to β-actin bands using scanning laser densitometry (GS-800 System, Bio-Rad, Hercules, CA, United States) and expressed as arbitrary units.

#### Quantitative Real Time Polymerase Chain Reaction (RT-PCR) Analysis of Insulin Receptor, RAGE, and TLR4 Gene Expression in the hippocampus

Briefly, extracted RNA (1 μg) was converted to complementary DNA using an RT-PCR kit (Stratagene, Cat. No. 600188, La Jolla, CA, United States) following the manufacturers’ directions. Then, SYBR Green JumpStart Taq ReadyMix (Sigma-Aldrich, Cat. No. S5193, St. Louis, MO, United States) was used to perform quantitative RT-PCR, in which complementary DNA (5 μl) was mixed with SYBR Green (12.5 μl), RNAse free water (5.5 µl), and the primer of each parameter (10 pmol/2 µl). The primer sequences are listed in Table 1. Then, PCR reactions were carried out, which included 40 cycles of denaturation at 95 °C for 15 s, annealing at 60 °C for 60 s, and extension at 72 °C for 60 s. To estimate the relative expression of target genes, the 2^−ΔΔCT^ formula was applied, using β-actin as a control.

**TABLE 1:** The primers sequences.

Gene	Forward primer	Reverse primer
β-Actin	5′-TAT​CCT​GGC​CTC​ACT​GTC​CA -3′	5′-AAC​GCA​GCT​CAG​TAA​CAG​TC-3′
Insulin receptor	5′-TTT​TCG​TCC​CCA​GGC​CAT​C-3′	5′-GTC​ACA​TTC​CCA​ACA​TCG​CC-3′
RAGE	5′-AGA​ACA​TCA​CAG​CCC​GGA​TT-3′	5′-TTC​CTG​TGT​TCA​GTT​TCC​AT-3′
TLR4	5′-CAA​CAT​CAT​CCA​GGA​AGG​C-3′	5′-GAA​GGC​GAT​ACA​ATT​CCA​CC-3′

#### Histopathological Examination

Directly after dissection, brains were fixed in 10% formalin for 24 h. Then they were washed, processed in serial grades of alcohol, and fixed in paraffin blocks. Sagittal brain sections (4 μm) were cut by rotatory microtome for demonstration of the cerebral cortex and hippocampal regions (the cornu ammonis (CA1, 2, 3 and 4) and the dentate gyrus (DG)) in different samples. Tissue sections were stained by Hematoxylin and Eosin (H and E), and Nissl stain and were examined by using light microscope.

### Statistical Analysis

To determine normality and homogeneity of variance, we performed Shapiro–Wilk and Brown–Forsythe tests, respectively, on all data. Data sets that met the assumptions of the parametric analysis were analyzed using one-way ANOVA followed by Tukey’s multiple comparisons test and were expressed as mean ± SD. The mean escape latency in the MWM was analyzed by two-way ANOVA. A probability level of less than 0.05 was considered statistically significant. Statistical analysis was carried out using GraphPad Prism software version 8 (San Diego, CA, United States).

## Results

### PFT Amended STZ-Induced Cognitive Deterioration

**FIGURE 1:**
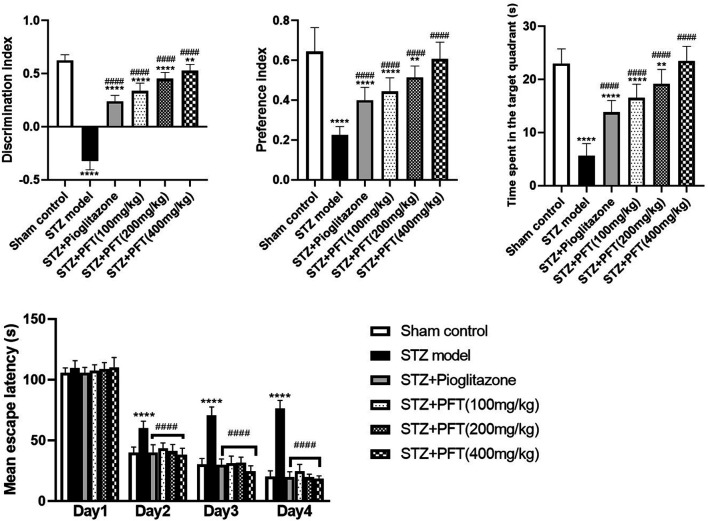
PFT amended STZ-induced cognitive deterioration. Each bar with vertical line represents the mean ± SD of twelve mice per group; ^**^significantly different from the sham control group at *p* < 0.01, ^****^significantly different from the sham control group at *p* < 0.0001, ^####^significantly different from the STZ group at *p* < 0.0001 using One-Way ANOVA followed by Tukey’s multiple comparisons test for discrimination index, preference index and the time spent in the target quadrant, while using Two-way ANOVA for the mean escape latency.

As compared with the sham control group, mice injected with STZ displayed marked impairment in memory and learning functions in the NOR test (*p* < 0.0001; Figure 1). Mice receiving PFT (100, 200, and 400 mg/kg) exhibited a substantial improvement in cognitive function in a dose-dependent manner, which was demonstrated by a considerable rise in the discrimination (4, 39.5, and 62.1%, respectively) and preference (97.0, 128.9, and 169.6%, respectively) indices as compared with the STZ-treated mice, *F* (5, 66) = 336.3 and 47.17 respectively (*p* < 0.0001). The effect of PFT was equivalent to that of pioglitazone, which significantly augmented the discrimination (by 26.2%) and preference indices (by 77.8%) as compared with the STZ group.

The mean escape latency in the MWM did not differ significantly between all groups on the first training day. However, beginning on the second day and until the fourth day, mice treated with PFT reached the platform faster than mice injected with STZ. On the test day, PFT-treated mice (100, 200, and 400 mg/kg) spent a markedly longer time than the STZ-injected mice in the aimed quadrant where the platform was previously sited (2.9-, 3.4-, and 4.1-fold, respectively), *F* (5, 66) = 81.3 (*p* < 0.0001). The effect of PFT was in line with that of pioglitazone (2.4-fold elevation as compared with the STZ group; Figure 1).

### PFT Overturned STZ-Induced Changes in Ach and Aβ_1–42_ Levels in the Hippocampus

**FIGURE 2:**
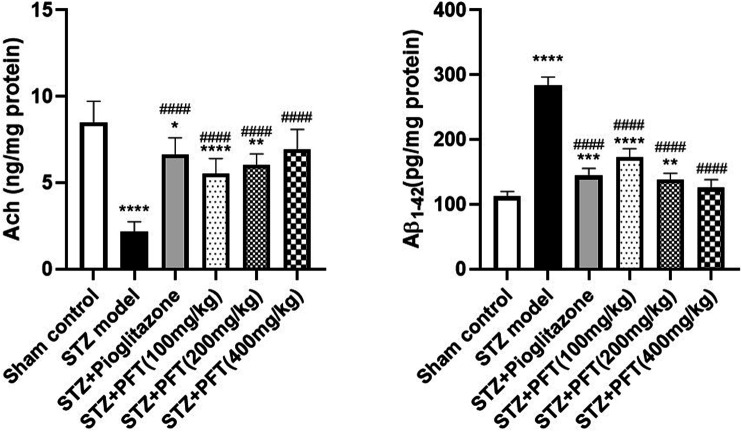
PFT overturned STZ-induced changes in Ach and Aβ_1-42_ levels in the hippocampus. Each bar with vertical line represents the mean ± SD of six mice per group; ^*^significantly different from the sham control group at *p* < 0.05, ^**^significantly different from the sham control group at *p* < 0.01, ^***^significantly different from the sham control group at *p* < 0.001, ^****^significantly different from sham control group at *p* < 0.0001, ^####^significantly different from the STZ group at *p* < 0.0001 using One-Way ANOVA followed by Tukey’s multiple comparisons test.

The Ach level greatly declined and the Aβ_1–42_ level was noticeably increased in the hippocampus of STZ-injected mice as compared with their counterparts in the sham control group, *F* (5, 30) = 31.5 and 180.2, respectively (*p* < 0.0001; Figure 2). In contrast, treatment with PFT (100, 200, and 400 mg/kg) obviously restored the hippocampal Ach level by 2.6-, 2.8-, and 3.2-fold, respectively, and repressed the Aβ_1–42_ level by 34.1, 45.3, and 52.5%, respectively, as compared with the STZ group (*p* < 0.001 and *p* < 0.0001). These results were comparable with those displayed by pioglitazone (elevated Ach level by 3.1-fold and suppressed Aβ_1–42_ level by 43.4% as compared with the STZ group).

### PFT Reversed STZ-Induced Variations in the Insulin Receptor Expression and IDE Level in the Hippocampus

**FIGURE 3:**
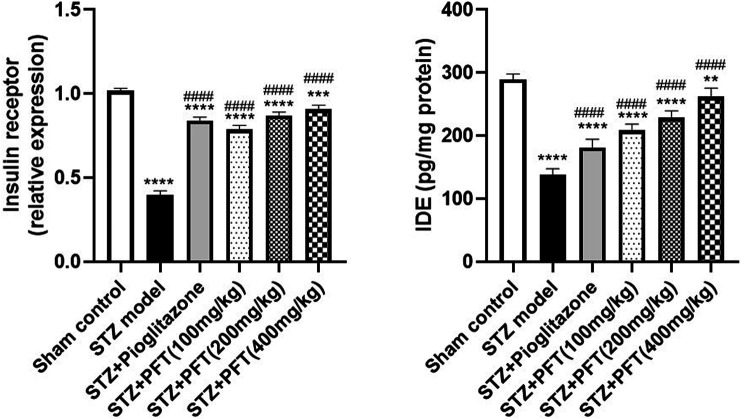
PFT reversed STZ-induced variations in the insulin receptor expression and IDE level in the hippocampus. Each bar with vertical line represents the mean ± SD of three mice per group for insulin receptor expression and of six mice per group for IDE level; ^**^ significantly different from the sham control group at *p* < 0.01, ^***^significantly different from the sham control group at *p* < 0.001, ^****^significantly different from the sham control group at *p* < 0.0001, ^####^significantly different from the STZ group at *p* < 0.0001 using One-Way ANOVA followed by Tukey’s multiple comparisons test.

Mice injected with STZ showed a noticeable repression in the expression of insulin receptors, *F* (5, 12) = 279.4 (*p* < 0.0001) and IDE level, *F* (5, 30) =143.8 (*p* < 0.0001) in the hippocampus as compared with the sham control mice (Figure 3). On the other hand, mice supplemented with PFT (100, 200, and 400 mg/kg) revealed a momentous elevation in insulin receptor expression (2.0-, 2.2-, and 2.3-fold, respectively) as well as the IDE level (1.5-, 1.7-, and 1.9-fold, respectively) as compared with the STZ-treated animals (*p* < 0.0001), an effect that was analogous to that of pioglitazone in STZ mice (2.1- and 1.3-fold, respectively).

### PFT Ameliorated STZ-Induced Discrepancies in p-PI3K, p-Akt, p-GSK-3β, p-mTOR, and p-tau Expression in the Hippocampus

**FIGURE 4:**
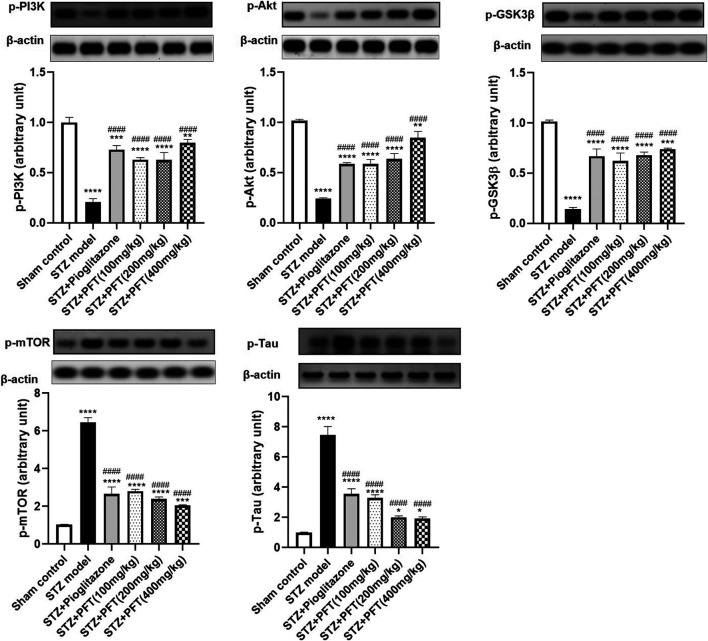
PFT ameliorated STZ-induced discrepancies in p-PI3K, p-Akt, p-GSK-3β, p-mTOR, and p-tau expression in the hippocampus. Each bar with vertical line represents the mean ± SD of three mice per group; ^*^significantly different from the sham control group at *p* < 0.05, ^**^significantly different from the sham control group at *p* < 0.01, ^***^ significantly different from the sham control group at *p* < 0.001, ^****^significantly different from the sham control group at *p* < 0.0001, ^####^significantly different from the STZ group at *p* < 0.0001 using One-Way ANOVA followed by Tukey’s multiple comparisons test.

STZ enormously decreased the expression of p-PI3K, p-Akt, and p-GSK-3β, whereas it immensely increased the expression of p-mTOR, and p-tau in the hippocampus as compared with the sham control group, *F* (5, 12) = 99.0, 127.5, 109.8, 283.7, and 184.3, respectively (*p* < 0.0001; Figure 4). However, PFT treatment (100, 200, and 400 mg/kg) significantly upregulated the expression of p-PI3K (3.0-, 3.0-, and 3.8-fold, respectively), p-Akt (2.4-, 2.7-, and 3.5-fold, respectively), and p-GSK-3β (4.3-, 4.7-, and 5.1-fold, respectively), and downregulated the expression of p-mTOR (56.6%, 62.8%, and 68.3, respectively), and p-tau (56.0, 73.2, and 74.3%, respectively) in the hippocampus as compared with the STZ group (*p* < 0.0001). These outcomes were in line with those of pioglitazone, which raised the expression of p-PI3k, p-Akt, and p-GSK-3β by 3.5-, 2.4- and 4.6-fold, respectively, whereas it suppressed the expression of p-mTOR, and p-tau by 71.0, 58.7, and 52.5%, respectively.

### PFT Diminished STZ-Induced Oxidative Stress

**FIGURE 5:**
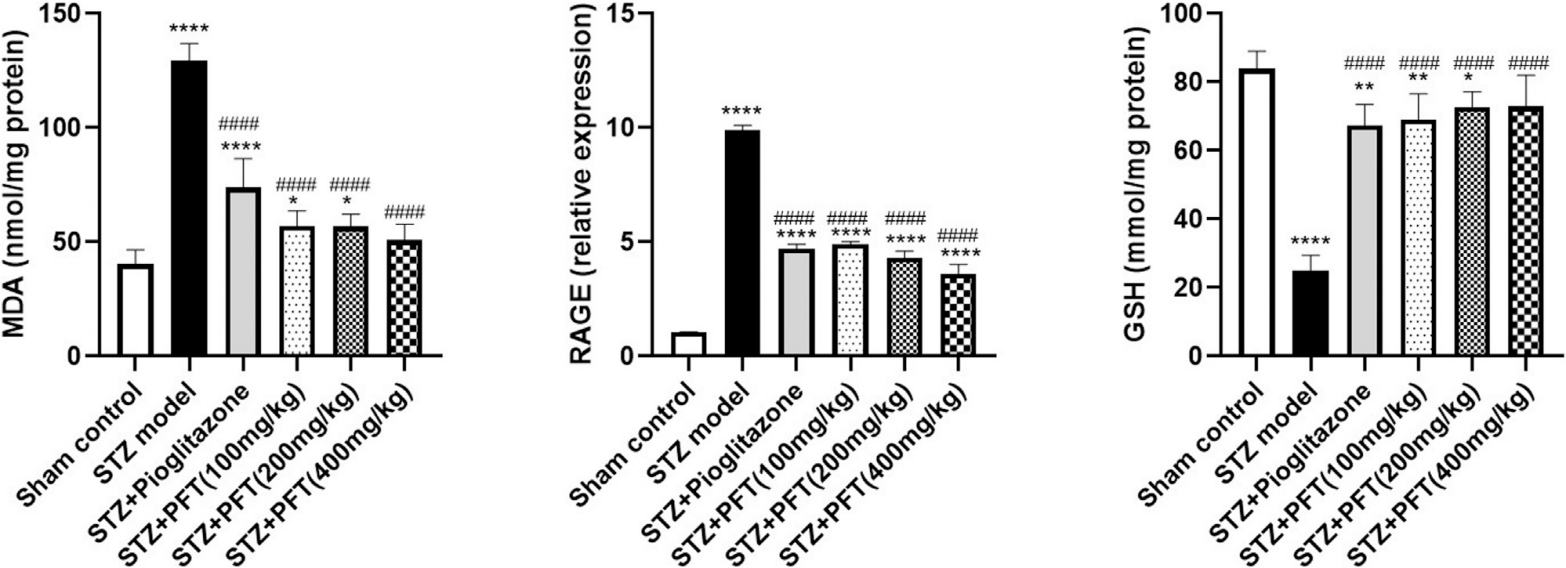
PFT diminished STZ-induced oxidative stress. Each bar with vertical line represents the mean ± SD of six mice per group for MDA and GSH and of three mice per group for RAGE; ^*^significantly different from the sham control group at *p* < 0.05, ^**^significantly different from the sham control group at *p* < 0.01, ^****^significantly different from the sham control group at *p* < 0.0001, ^####^significantly different from the STZ group at *p* < 0.0001 using One-Way ANOVA followed by Tukey’s multiple comparisons test.

Animals administered STZ showed a striking increasing in MDA level and RAGE expression, *F* (5, 30) = 97.71 and *F* (5, 12) = 432.2, respectively (*p* < 0.0001) along with a drop in GSH activity, *F* (5, 30) = 63.0 (*p* < 0.0001) in the hippocampus as compared with the sham control animals (Figure 5). However, animals treated with PFT (100, 200, and 400 mg/kg) displayed a remarkable decline in MDA level by 56.0, 56.2, and 60.8%, respectively, and a decline in RAGE expression by 50.4, 56.5, and 63.5%, respectively, together with enhancement of GSH activity by 2.8-, 2.9-, and 2.9-fold, respectively, as compared with STZ-exposed animals (*p* < 0.0001). These effects were parallel to those of pioglitazone, which diminished the MDA level and RAGE expression by 43.0% and 52.5%, respectively, and augmented GSH activity by 2.7-fold in STZ animals.

### PFT Alleviated STZ-Induced Inflammatory Changes

**FIGURE 6:**
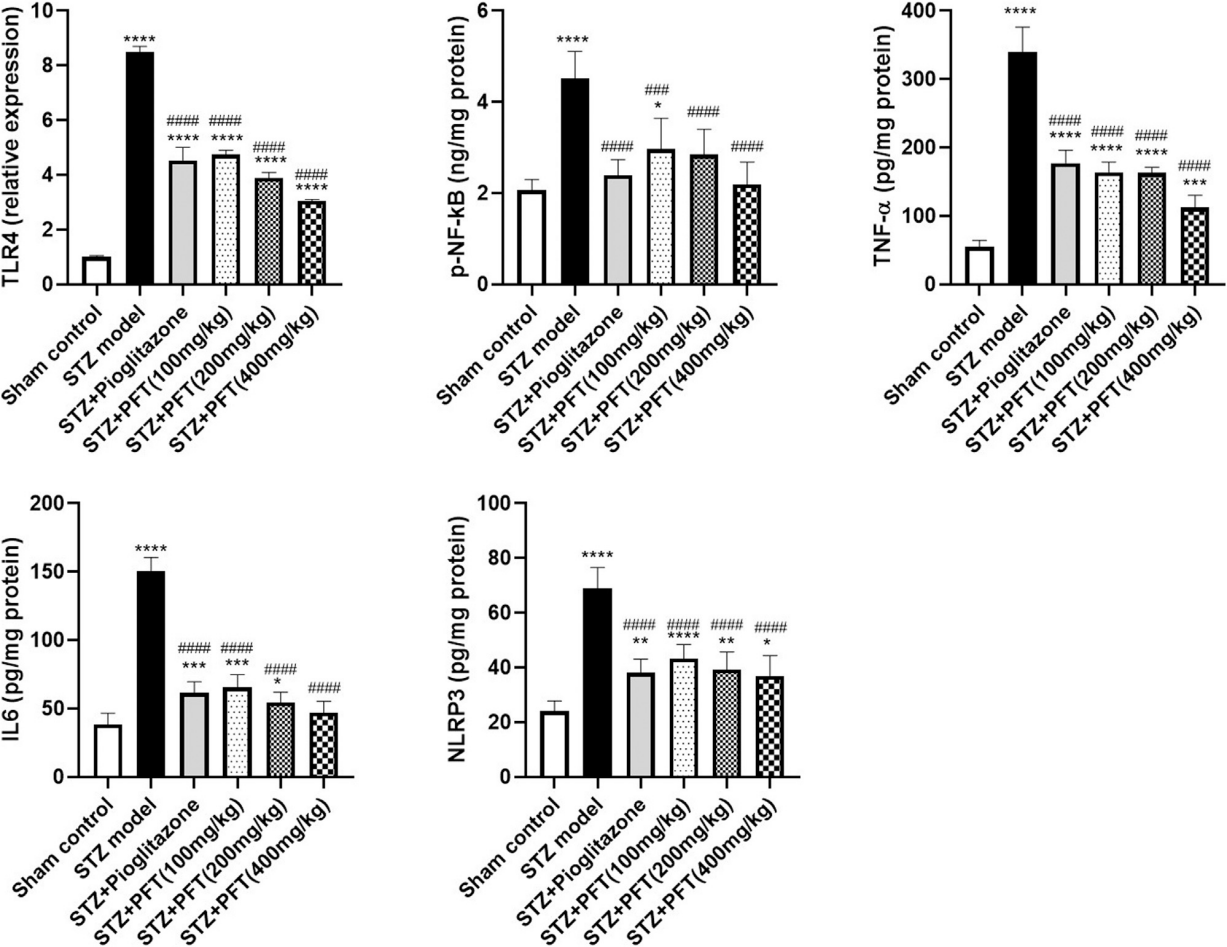
PFT alleviated STZ-induced inflammatory changes. Each bar with vertical line represents the mean ± SD of three mice per group for TLR4 and of six mice per group for p-NF-kB, TNF-α, IL6, and NLRP3; ^*^significantly different from the sham control group at *p* < 0.05, ^**^significantly different from the sham control group at *p* < 0.01, ^***^significantly different from the sham control group at *p* < 0.001, ^****^significantly different from the sham control group at *p* < 0.0001, ^###^significantly different from the STZ group at *p* < 0.001, ^####^significantly different from the STZ group at *p* < 0.0001 using One-Way ANOVA followed by Tukey’s multiple comparisons test.

As compared with the sham control group, the group injected with STZ demonstrated a noticeable increase in various inflammatory markers, including TLR4, p-NF-κB, TNF-α, IL6, and NLPR3, in the hippocampus, *F* (5, 12) = 336.8, *F* (5, 30) = 19.3, 138.5, 132.6, and 35.0, respectively (*p* < 0.0001; Figure 6). As compared with the STZ group, the group receiving PFT supplementation (100, 200, and 400 mg/kg) showed a reduction in the expression of TLR4 by 44.1, 54.2, and 64.0%, respectively; p-NF-κB by 34.4, 37.0, and 51.6%, respectively; TNF-α by 51.7, 51.9, and 66.8%, respectively; IL6 by 56.5, 63.7, and 68.6%, respectively; and NLRP3 by 37.0, 42.9, and 46.4%, respectively *(p* < 0.0001). The effect of PFT on these indicators corresponded to that of pioglitazone, which decreased TLR4 by 46.6%, p-NF-κB by 47.1%, TNF-α by 47.7%, IL6 by 59.0%, and NLRP3 by 44.6%.

### PFT Preserved the Cerebral Cortex and Hippocampal Neurons From STZ-Induced Degeneration

**FIGURE 7:**
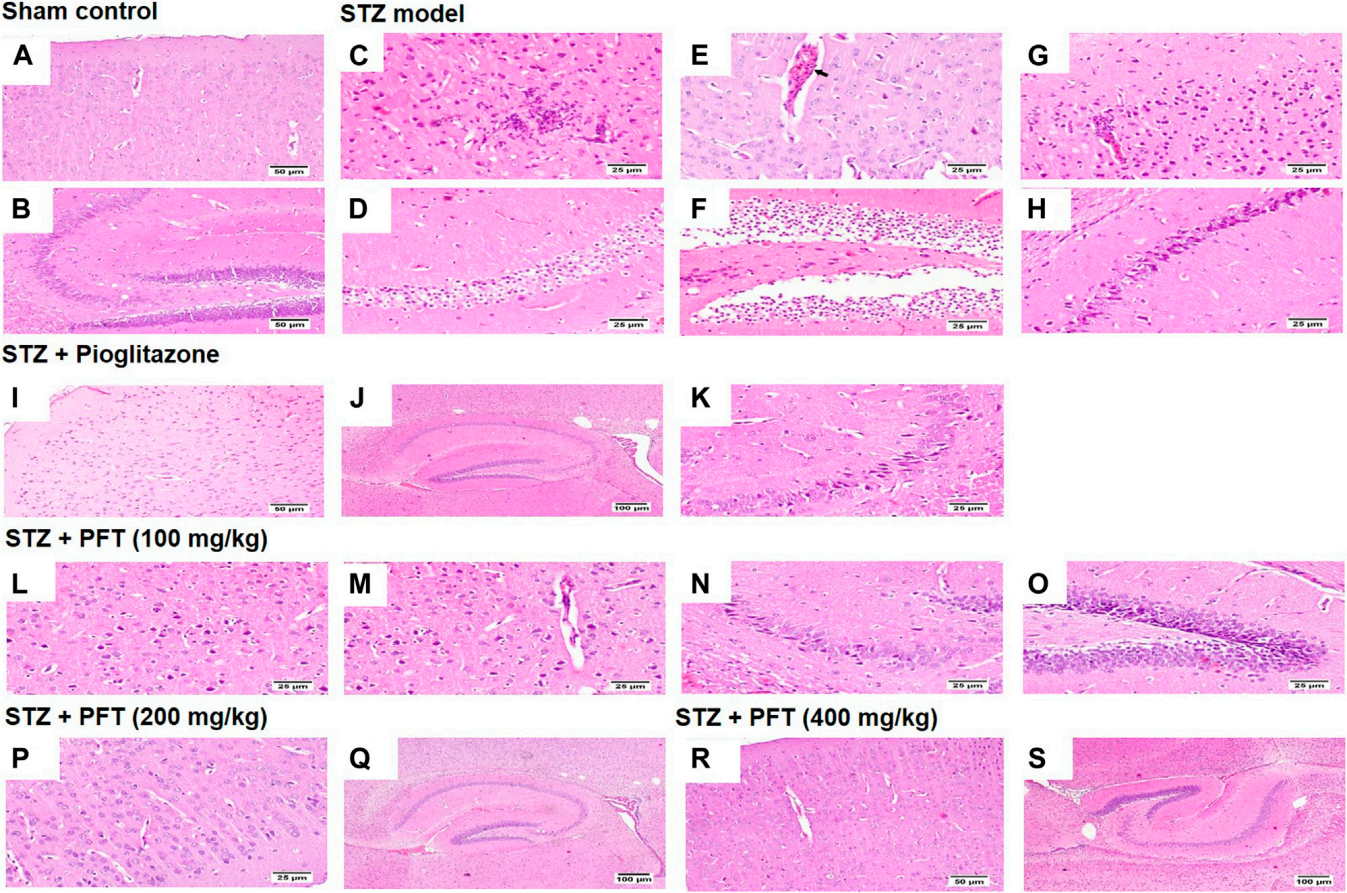
Histological sections of H and E staining in the cerebral cortex and hippocampus of the experimental groups; where sham control mouse showed normal histology of neurons in the cerebral cortex **(A)**, and the hippocampus **(B)**. STZ model group brain showed area of malacia associated with focal gliosis and necrosis **(C)**, thickened blood vessel wall (arrow) **(E)**, and perivascular lymphocytic cuffing with surrounding dark degenerated neurons in the cerebral cortex **(G)**, beside neuronal edema of CA1 and CA2 regions **(D)**, as well as neuronal edema of DG region **(F)** and shrunken dark degenerated neuron in CA3 of the hippocampus **(H)**. Mice receiving pioglitazone showed apparently normal neurons in the cerebral cortex **(I)** and apparently normal nerve cells in most of the hippocampus regions **(J)** with few scattered dark degenerated neurons in CA3 and CA4 regions **(K)**. PFT (100 mg/kg) brain revealed variable number of dark degenerated neurons accompanied by gliosis in the cerebral cortex **(L)** along with neuronal degeneration and neuronophagia with mild thickening of blood vessel wall **(M)**, besides, dark degenerated neurons in the CA4 region **(N)**, and in the DG region of the hippocampus **(O)**. Brain of PFT (200 mg/kg) apparently normal neurons in the cerebral cortex **(P)**, and apparently normal neurons in the hippocampus with few scattered neurons in CA4 and DG **(Q)**. Brain of PFT (400 mg/kg) displayed apparently normal neurons cerebral cortex **(R)**, and apparently normal neurons in the hippocampus **(S)**.

Histopathological examination with H and E staining showed a normal histological structure of the brain tissue of the sham control group. In contrast, the STZ model group exhibited diffuse gliosis in the cerebral cortex with the existence of necrotic debris along with thickening of the wall of the blood vessels and perivascular lymphocytic infiltration associated with numerous scattered dark degenerated neurons and infiltration of inflammatory cells. The striatum and the hippocampus displayed severe widespread edema with dark deteriorated neurons in the CA3 and CA4 hippocampal regions. PFT treatment at a dose of 100 mg/kg partially improved STZ-induced histological deterioration, whereby the cerebral cortex showed moderate numbers of shrunken degenerated neurons with gliosis and neuronophagia with mild thickening of the blood vessel walls, and the hippocampus presented some dark neurons in the CA1, CA3, and DG regions. Meanwhile, an increase in the dose to 200 and 400 mg/kg protected against the deleterious effect of the STZ injection, which showed apparently normal microscopic structure of the brain tissue in the cerebral cortex and various areas of the hippocampus, except for few degenerated cells in CA4 and DG observed with the 200-mg/kg dose. The protective effect exerted by PFT treatment was comparable with that of pioglitazone, which ameliorated the histopathological alterations induced by STZ and showed apparently normal neurons in the cerebral cortex with individual dark deteriorated neurons in the CA3 and C4 regions of the hippocampus (Figure 7).

**FIGURE 8:**
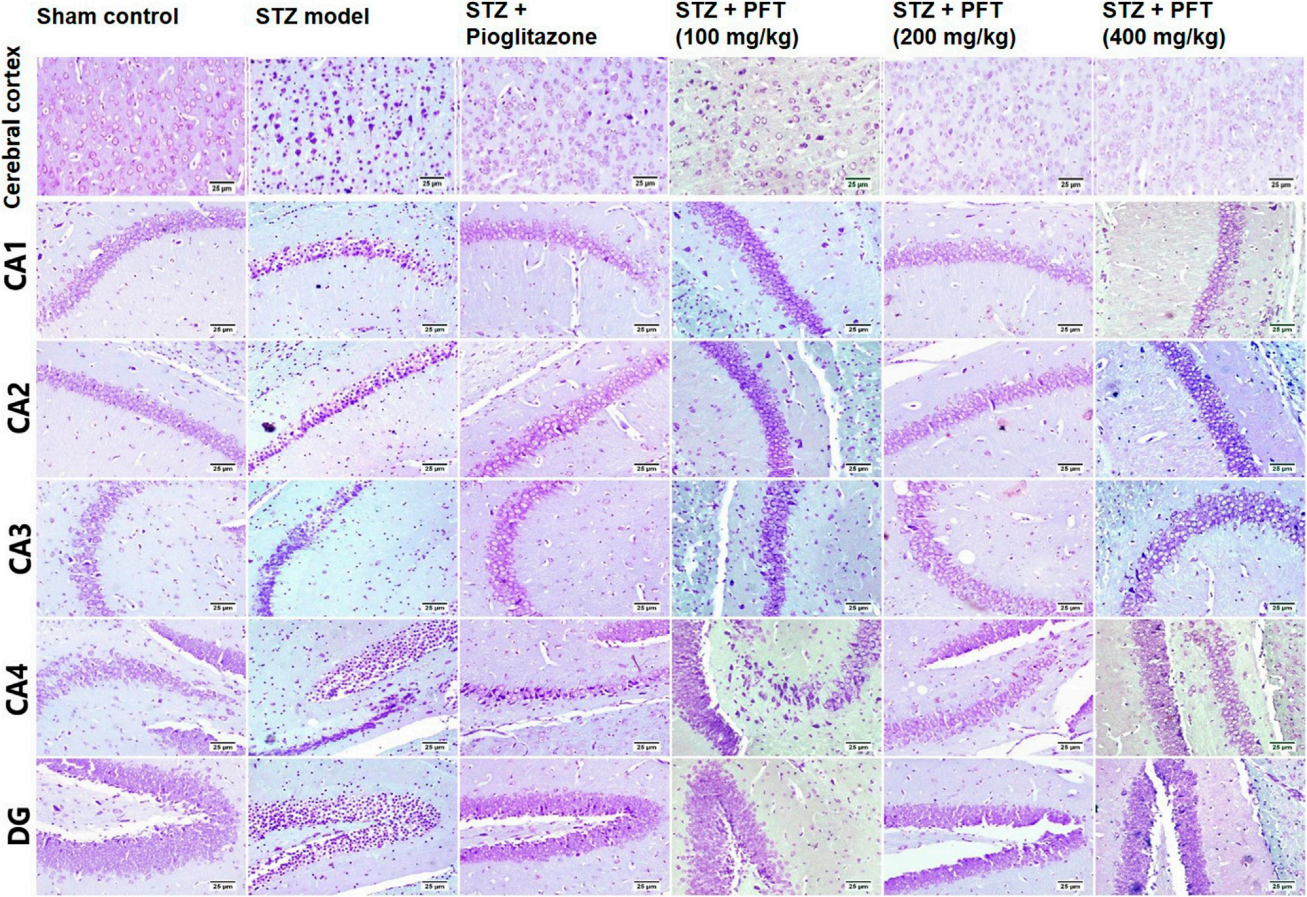
Nissl staining of the cerebral cortex, and the hippocampal regions (CA1, CA2, CA3, CA4 and DG) sections. Sham control group showed normal intact neurons in the cerebral cortex and in all the hippocampus region. Variable number of shrunken neurons with pyknotic nuclei was detected among different treated groups with evidence of increase in STZ group.

In addition, we evaluated the rate of survival by calculating the percentage of intact neurons after staining with Nissl stain (Table 2). As compared with the sham control group, the STZ group demonstrated a significant decrease in the survival rate of the cerebral cortex and the hippocampal regions (CA1, CA2, CA3, CA4, and DG) *F* (5, 30) = 612.7, 3704, 2342, 1420, 1789, and 1476, respectively (*p* < 0.0001). On the other hand, PFT treatment (100, 200, and 400 md/kg) highly preserved the neurons in the cerebral cortex (4.0-, 3.9-, and 4-fold, respectively) and the hippocampus (CA1: 3.0-, 3.1-, and 3.1-fold, respectively; CA2: 4-, 4.1-, 4.1-fold, respectively; CA3: 2.5-, 2.6-, and 2.6-fold, respectively; CA4: 2.5-, 3-, and 3-fold, respectively; DG: 3.7-, 3.9-, and 3.8-fold, respectively) in comparison with the STZ group (Figure 8). The activity of PFT resembled that of pioglitazone, which conserved neurons in the cerebral cortex by 3.5-fold and in CA1, CA2, CA3, CA4, and DG by 3.1-, 4.1-, 2.6-, 2.6-, and 3.8-fold, respectively.

**TABLE 2:** The percent of survival neurons of mice brain in the cerebral cortex and various area of the hippocampus that recorded in different experimental groups.

Group	Survival rate (%)					
	Cerebral cortex	CA1	CA2	CA3	CA4	DG
Sham control	98.2 ± 1.7	97.5 ± 1.1	98.9 ± 1.0	97.8 ± 1.3	99.11 ± 1.0	96.6 ± 0.7
STZ model	23.4 ± 5.3^****^	31.0 ± 1.0^****^	23.3 ± 1.6^****^	35.4 ± 2.3^****^	30.9 ± 1.3^****^	23.9 ± 3.2^****^
STZ + Pioglitazone	79.7 ± 2.5^****####^	95.7 ± 0.9^####^	94.6 ± 1.2^***####^	91.6 ± 1.1^****####^	79.6 ± 1.2^****####^	90.4 ± 1.4^****####^
STZ + PFT (100 mg/kg)	91.1 ± 1.5^**####^	93.9 ± 0.9^****####^	91.6 ± 1.4^****####^	89.9 ± 0.9^****####^	76.1 ± 1.4^****####^	88.5 ± 1.4^****####^
STZ + PFT (200 mg/kg)	89.5 ± 1.3^****####^	95.3 ± 1.3^*####^	94.5 ± 1.5^***####^	90.8 ± 0.9^****####^	92.5 ± 2.0^****####^	92.3 ± 1.5^***####^
STZ + PFT (400 mg/kg)	91.8 ± 2.1^**####^	95.8 ± 1.1^####^	95.5 ± 2.0^**####^	93.3 ± 2.0^****####^	94.0 ± 1.5^****####^	91.3 ± 1.3^**####^

Data of survival rate represents the mean ± SD of three mice per group; ^*^ significantly different from the sham control group at *p* < 0.05, ^**^significantly different from the sham control group at *p* < 0.01, ^***^significantly different from the sham control group at *p* < 0.001, ^*****^significantly different from the sham control group at *p* < 0.0001, ^####^significantly different from the STZ group at *p* < 0.0001 using One-Way ANOVA followed by Tukey’s multiple comparisons test.

## Discussion

This study demonstrated the favorable effect of the modulation of gut microbiota and attenuation of insulin resistance via PFT in ameliorating various pathological mechanisms that underlie the pathogenesis of AD with consequent improvement in cognition. PFT not only enhanced the level of hippocampal Ach but also reduced Aβ_1-42_ content and preserved neurons in the hippocampus and the cerebral cortex in an STZ model of AD in a dose-dependent manner. In addition, PFT improved insulin signaling, which was proven by the upregulation of insulin receptor expression and augmentation of PI3K/Akt signaling with subsequent modulation of GSK-3β and mTOR activity, which eventually reduced the accumulation of p-tau. Moreover, PFT significantly diminished oxidative stress and inflammation with further protection against neurodegeneration. These remarkable effects were comparable with that produced by pioglitazone, which was proven to diminish cognitive impairment along with neuronal preservation via enhancement of insulin signaling, in addition to displaying antioxidant and anti-inflammatory activities (Sato et al., 2011; Mandrekar-Colucci et al., 2012; Searcy et al., 2012).

In the present study, we used STZ to induce a sporadic AD model in mice via a single ICV injection, which has been proven to be a valuable model for studying neuroprotective therapies (Gao et al., 2014). Herein, STZ-treated mice presented severe cognitive dysfunction, alongside the depletion of Ach in the hippocampus and elevation of Aβ_1–42_, with the accumulation of hyperphosphorylated tau and neuronal death. This was in agreement with the findings of previous studies (Kosaraju et al., 2013; Hira et al., 2019).

Remarkably, insulin resistance has been shown to be immensely implicated in AD pathology (Talbot et al., 2012; Gabbouj et al., 2019). This was based on the results of several studies that proved the association between impaired insulin signaling and the development of dementia in AD, in addition to evidence of suppressed brain insulin receptors and signaling in AD patients (Steen et al., 2005; Liu et al., 2011; Butterfield et al., 2014). Moreover, insulin was shown to play a vital role in the brain via binding to its receptor, which is found abundantly in the hippocampus and the cerebral cortex, the core brain areas that are disturbed in AD pathology (Plum et al., 2005; Freude et al., 2009). Upon binding to its receptor, insulin promotes the recruitment of insulin receptor substrate-1, which in turn activates the PI3K/Akt pathway that regulates neuronal development and plasticity, as well as learning and memory processes (Plum et al., 2005; Horwood et al., 2006). Thus, impaired insulin signaling in the brain is associated with neuronal death and cognitive dysfunction (Bomfim et al., 2012; Talbot et al., 2012). It is interesting to note that insulin resistance and subsequent hyperinsulinemia, which occur in metabolic syndrome, are implicated in Aβ aggregation and p-tau accumulation (Razay et al., 2007; Gabbouj et al., 2019). For Aβ aggregation, hyperinsulinemia results in the downregulation of insulin receptors with consequent alteration in insulin signaling in the brain, which in turn elevates the generation of Aβ and decreases its clearance by hampering the production of the enzyme required for its degradation, IDE, as well as competing with Aβ for it (Zhao et al., 2004; Luchsinger, 2008). For p-tau accumulation, reduced insulin activity results in impaired PI3K/Akt signaling, which controls GSK-3β and mTOR downstream factors, which are the key regulators of tau hyperphosphorylation (Kitagishi et al., 2014; Cai et al., 2015). GSK-3β is the chief kinase that phosphorylates tau, it is constitutively active and inhibited by phosphorylation at Ser 9. Reduced PI3K/Akt signaling results in a decrease in the phosphorylation state of GSK-3β, thus stimulating its activity and promoting tau hyperphosphorylation (Takashima, 2006). Moreover, mTOR signaling is augmented by suppressed PI3K/Akt activity, which also endorses tau hyperphosphorylation via the inhibition of autophagy and protein-phosphatase-2A activity required for restraining the level of hyperphosphorylated tau (Meske et al., 2008; Chong et al., 2012; Cai et al., 2015). In addition, mTOR promotes GSK-3β activation with further augmentation of hyperphosphorylated tau (Meske et al., 2008). Collectively, the regulation of insulin activity may be a promising intervention for halting AD progression (Gabbouj et al., 2019). In particular, the dysfunction of the gut microbiome was stated to be associated with insulin resistance and was proven to be linked to metabolic syndrome, which mainly constitutes insulin resistance and hyperinsulinemia (Al-Qahtani et al., 2014; Wang et al., 2020). Thus, the modulation of gut microbiota may hinder insulin resistance and improve AD symptoms. In this context, PFT enhanced the insulin activity in the brain of animals, which was confirmed by upregulation of insulin receptors and IDE in the hippocampus, along with restoration of PI3K/Akt signaling, with subsequent modulation of GSK-3β and mTOR activity. The enhancement of insulin signaling by PFT resulted in a decrease in the level of Aβ_1–42_ protein and a hindrance to the accumulation of p-tau, together with preservation of neurons in the hippocampus and the cerebral cortex against STZ-induced death. These activities led to the restoration of hippocampal Ach level and consequent cognition improvement revealed in the NOR and MWM tests. These beneficial effects exerted by PFT are similar to those of pioglitazone, which was previously shown to improve insulin sensitivity, enhance cognitive function, and reduce the hippocampal Aβ_1–42_ protein level and p-tau deposits (Gupta and Gupta, 2012; Mandrekar-Colucci et al., 2012; Searcy et al., 2012).

In addition to the direct effect of insulin resistance on Aβ accumulation and tau hyperphosphorylation, impaired glucose metabolism predisposes mitochondrial dysfunction with sequential oxidative stress, which are critically linked to the development of AD (Carlsson, 2010; Butterfield et al., 2014). Briefly, reduced insulin activity is associated with reduced glucose metabolism that promotes mitochondrial damage and diminished energy production together with a massive increase in reactive oxygen species (ROS) that depletes the antioxidant capacity (Butterfield, 2006; Butterfield et al., 2014). Moreover, ROS exhibit oxidative damage of proteins, nucleic acids and lipids, which are associated with the progression of AD (Butterfield et al., 2001; Di Domenico et al., 2010). It is worth noting that the oxidation of a protein’s amino acids results in the production of AGE, which is a distinctive pathological feature of AD (Smith et al., 1995; Sasaki et al., 1998; Butterfield et al., 2014). Previous studies revealed that AGE hastens the accumulation of Aβ through the generation of free radicals that promote Aβ protein misfolding and aggregation, as well as by enhancing the stabilization of neurofibrillary tangles via glycation of tau (Zhao and Townsend, 2009; Butterfield et al., 2014). In this context, an elevated AGE level was found in AD amyloid plaques and neurofibrillary tangles (Vitek et al., 1994; Smith et al., 1995). Furthermore, RAGE was upregulated in the brains of AD patients (Valente et al., 2010). RAGE was discovered to promote additional Aβ accumulation by increasing the transportation of Aβ across the blood-brain barrier, in addition to acting as a receptor for Aβ protein and hence inducing Aβ-mediated microglial activation and consequent inflammation, which are linked to the pathogenesis of AD (Lue et al., 2001; Chaney et al., 2005). Taken together, the resolution of insulin resistance inhibits oxidative stress-induced damage and neuronal death. Indeed, oxidative stress was found to result from dysbiosis, because the gut microbiome is charged in the production of antioxidants, yet its alteration results in propagation of amyloids and lipopolysaccharides that stimulate ROS and consequent oxidative stress (Pistollato et al., 2016; Angelucci et al., 2019). Moreover, dysbiosis is related to hyperinsulinemia and hyperglycemia associated with metabolic syndrome, which represents a high-risk factor for AD development; therefore, the restoration of gut microbiota may inhibit oxidative stress and halt the progression of AD (Al-Qahtani et al., 2014; Wang et al., 2020). In this study, STZ resulted in obvious oxidative damage to the animals’ brains; however, PFT significantly decreased this oxidative stress, as evidenced by the decreased level of MDA, a lipid peroxidation marker, as well as the expression of RAGE, besides the increased activity of the antioxidant enzyme GSH. This substantial antioxidant activity exerted by PFT was paralleled by the observed neuronal preservation. The antioxidant activity of PFT was comparable with that of pioglitazone, which was reported to significantly augment the antioxidant defense with consequent cognitive enhancement in animals showing insulin resistance (Yin et al., 2013).

Certainly, insulin resistance is also charged in the production of inflammation (Barzilaym and Freedland, 2003; Finch and Morgan, 2007). Notably, inflammation is one of the chief features of AD, in which elevated levels of microglial activation and inflammatory markers such as TNF-α and interleukins are detected in the brains of AD patients (Minter et al., 2016; Bagyinszky et al., 2017). Previous researchers have verified that the decline in the PI3K/Akt pathway following insulin resistance leads to microglial activation and stimulation of the NF-kB signal, which provokes the generation of proinflammatory cytokines such as TNF-α and IL6 (Xu et al., 2010; Xu et al., 2018). In particular, TNF-α aggravates insulin resistance via activation of c-Jun kinase, which inhibits the phosphorylation of insulin receptor substrate-1 and hence blocks insulin signaling, resulting in exacerbation of the inflammatory response (Gregor and Hotamisligil, 2011). Moreover, enhancement of the NF-kB transcription promotes the activation of the NLRP3 inflammasome, which enhances the secretion of inflammatory cytokines with further stimulation of microglial activation, leading to a vicious cycle that eventually results in neuronal death (Tan et al., 2013). Interestingly, alteration of the gut microbiota causes the release of lipopolysaccharides that vastly activate TLR4, causing the inhibition PI3K/Akt signaling with subsequent activation of NF-kB and release of proinflammatory cytokines (Zhao et al., 2014; Zhao and Lukiw, 2015; Marques et al., 2016). In addition, dysbiosis is associated with metabolic syndrome, which has been proved to decrease insulin signaling and increase microglial proliferation, leading to progressive neurodegeneration in AD (Barzilaym and Freedland, 2003; Wang et al., 2020). To this end, modulation of the gut microbiota will deplete inflammation with successive neuronal preservation in AD. Consistent with this view, PFT effectively hampered STZ-induced neuroinflammation and protected the neurons in the hippocampus and the cerebral cortex from inflammation-induced death. The restoration of the gut microbiome by PFT leads to a significant downregulation in the expression of TLR4, which, with improvement of insulin signaling, leads to the enhancement of PI3K/Akt activity that suppresses the activity of NF-κB and the level of its downstream effectors with consequential amelioration of AD pathology. The favorable anti-inflammatory effect exerted by PFT was parallel to that of pioglitazone, which was shown to suppress inflammation with resultant attenuation of AD neurodegeneration (Fernandez-Martos et al., 2017).

In conclusion, PFT might be a potential therapy for AD, because it significantly decreases insulin resistance with subsequent oxidative stress and inflammation, thus targeting multiple mediators incorporated in the pathogenesis of AD. Moreover, we found that PFT substantially decreased Aβ_1-42_ protein accumulation and tau hyperphosphorylation and prevented neurodegeneration, effects that translated into an improvement in cognitive function. These favorable effects were exerted via the PFT’s ability to modulate the gut microbiota, which precipitates insulin resistance in addition to being implicated in metabolic syndrome, a crucial risk factor for the development of AD.

## Data Availability

The original contributions presented in the study are included in the article, further inquiries can be directed to the corresponding author.
